# Supplementary figures and images for: Latent representation of H&E images retains biological information in a breast cancer cohort
Source: PLoS One. 2025 Sep 25;20(9):e0329221. doi: 10.1371/journal.pone.0329221 (PMC12463230; doi:10.1371/journal.pone.0329221)

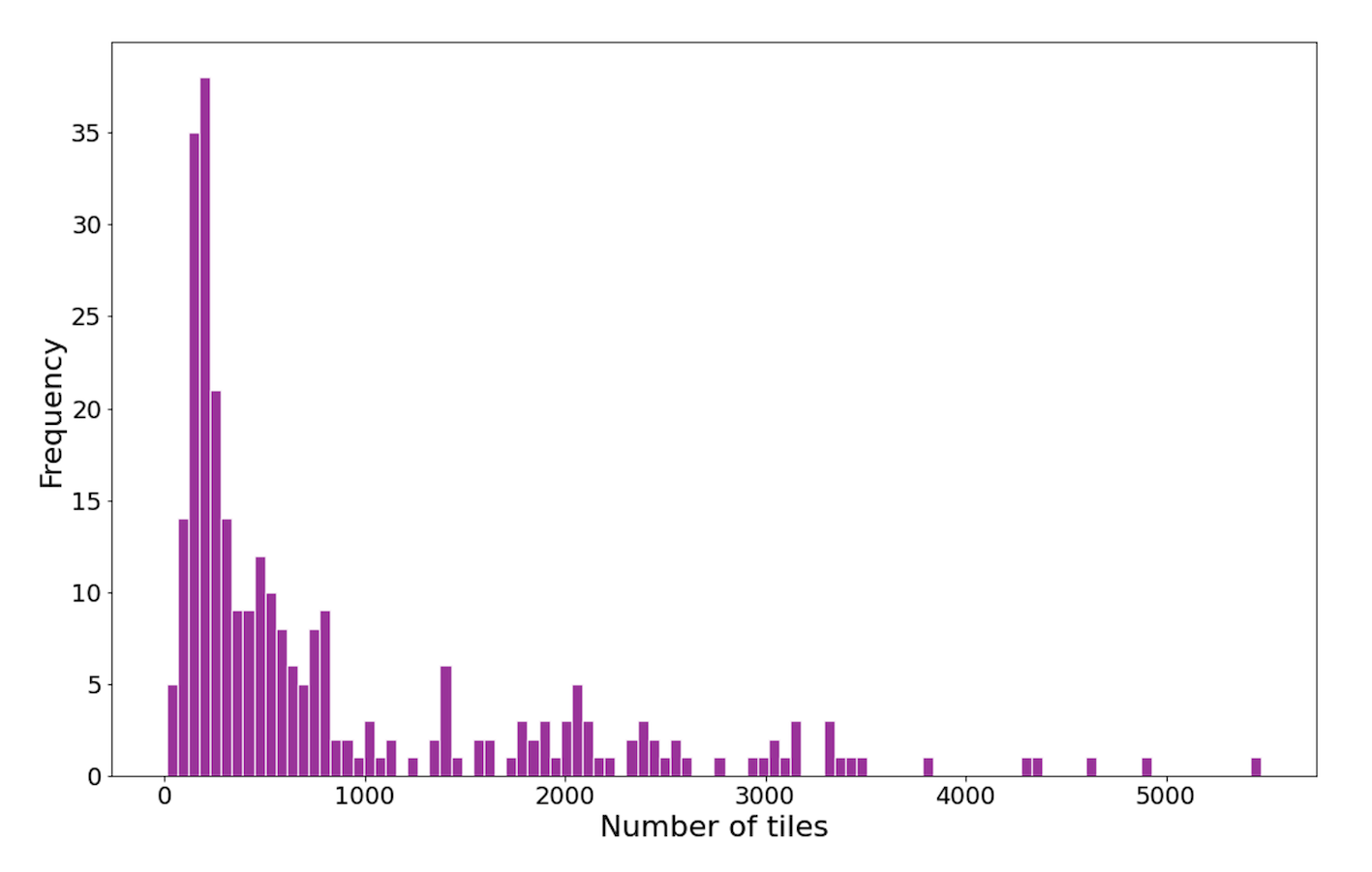

Supplement: S1 Fig — We analyzed a total of 283 images from 106 patients. The number of tiles varies due to the characteristics of the H&E staining and the microscopy. Namely - white space in the H&E images is ignored and not included in the tiles. (TIF) [file pone.0329221.s001.tif]

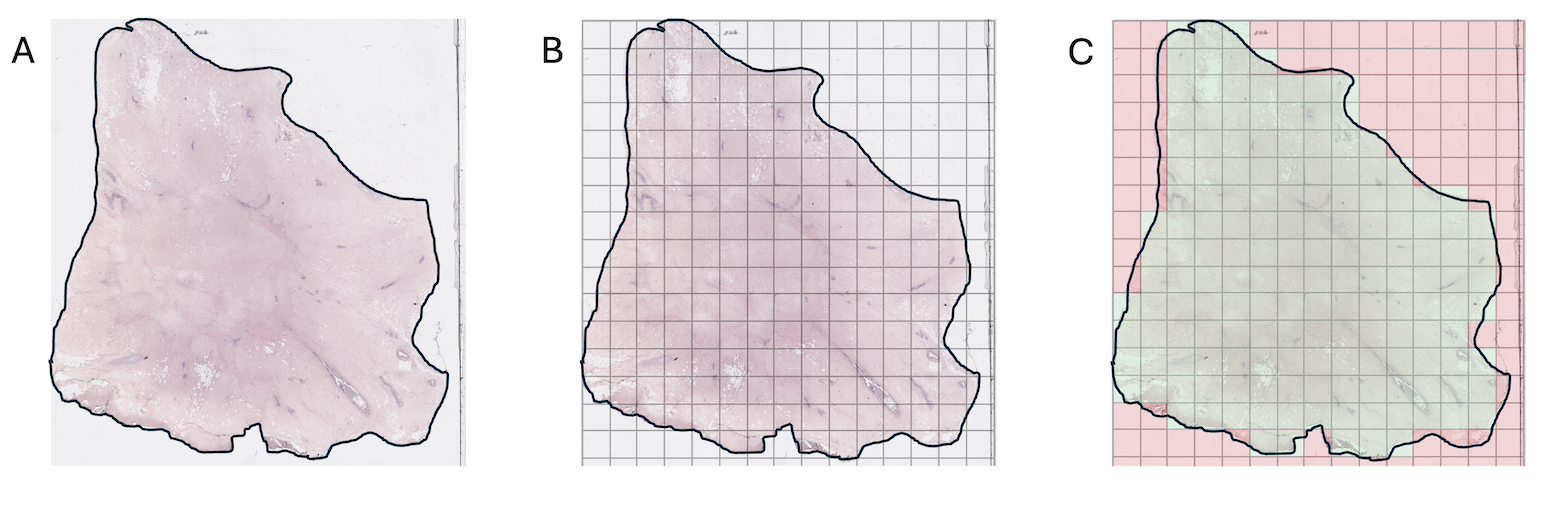

Supplement: S2 Fig — (A) Original biopsy image with the tissue outlined in black for better visualization. (B) Demonstration of the image tiling process (note: the actual tile size is significantly smaller in practice). (C) Tiles highlighted in green are selected, while those in red are discarded (containing more than 50% background). (TIF) [file pone.0329221.s002.tif]
