# Supplementary material for: Latent representation of H&E images retains biological information in a breast cancer cohort
Source: PLoS One. 2025 Sep 25;20(9):e0329221. doi: 10.1371/journal.pone.0329221 (PMC12463230; doi:10.1371/journal.pone.0329221)
Supplement: Attachment — Submitted filename: Response to Reviewers.pdf [file pone.0329221.s004.pdf]

Reichman University

Yakhini Research Group
Arazi School of Computer Science
Reichman University

Dear Editors of PLOS ONE,

Thank you for reviewing and handling our manuscript titled "LATENT REPRESENTATION OF H&E IMAGES RETAINS BIOLOGICAL INFORMATION IN A BREAST CANCER COHORT". Below, please find our response to all comments.

Reviewer 1:

Comment:
The authors describe a computational approach to classify regions of Haematoxylin and eosin stained Whole Slide Images of breast cancer with corresponding biological/molecular labels and to use autoencoders to generate a latent (lower dimension) representation of the data, highlighting how this impacts retention of clinically relevant information. The study is relevant and the approach appears to be sound but I suggest that the term "clinical" in the title be replaced as the majority of the data appears to be directly tumour-related (pathological/molecular or biological).

Response:
Thank you for this comment, we followed the suggestion and changed clinical to biological.

Comment:
In areas the language is somewhat unclear e.g. lines 273/274 making it difficult to understand. The paper would benefit from some language revision of the text in areas to improve readability and understanding.

Response:
Indeed, we went through the manuscript again and tried to modify the language. This includes the originally not very clear language in lines 273/274 as pointed out. Thank you for this comment.

Reviewer 2:

Comment:
#While an investigation into the effects of dimensionality reduction could be valuable to better understand prediction-associated features I believe this presented work only superficially explores this topic.

Response:
We agree that this is a first step in this research direction, but still think that the manuscript contains a sufficient volume of information and findings to be of interest to the community

Comment:
#The data towards assessing heterogeneity is PAM50 subtype tile-level predictions and values for patient-level model accuracy. It is difficult to make any conclusions from predictions of one label shown for 6 slides. While characterizing the heterogeneity of a tumor is valuable and deep learning applied to readily available H&E scanned slides would be an exciting tool for gaining such valuable information, this paper does not generate results towards this end. Ground truth spatial labels would be needed to evaluate whether this work does "support the assessment of heterogeneity" [346].

Response:
We thank the reviewer for this comment. In general, the lack of large dataset that can compare h&e to special transcriptomics is currently an obstacle to rigorously testing the potential of inferring the heterogeneity from h&E. That said, we believe that our results do suggest that this is potentially possible in the following sense. First, figure (annotation of pathologist) shows comparison of tile level classification to spatial annotation. Second, it is quite clear that if tile level classification is really accurate, then it can be used to infer heterogeneity. Of course, the latter is true only for heterogeneity based on the predicted traits. For example, in Alona et al [https://www.nature.com/articles/s41598-020-75708-z], the authors show that heterogeneity based on Ki67 and miR17, as inferred from h&e affects prognosis. We hope that future work can further support the validity of this approach, beyond just a few traits.
We changed the text in the discussion to be less general and more accurately reflects this point.

Comment:
#The authors note that their motivation for using an autoencoder to reduce the dimensionality of the data is to lessen the compute resources required to train classification models [227]. While it does take substantial compute resources to train large classification models, the most performant models in the field are massive multi-institutional foundation models. Therefore, hospitals implementing classification models for patient care would only be evaluating models for the patient at hand, a relatively trivial computational task. Additionally, the degradation of performance with dimensionality reduction is a significant concern for the clinical setting where the accuracy of tests is paramount. I feel that this is not the proper motivation for the subsequent work.

Response:
Agreed, we removed reference to computational resources and changed the opening paragraph of the section of autoencoders.

Comment:
#Ki67 is the only biomarker with results reported that allow for critical evaluation of how dimensionality reduction impacts performance. The performance for all labels should be reported.

Response:
We chose to focus on KI67 because, first, it is a very important prognostic marker, and second, literature has already shown its association to morphology. It therefore serves as a baseline case for our investigation. We feel like including the other biomarkers will overload the paper. It also requires more label specific training. A survey of more biomarkers is possibly a good topic for a follow-up paper. We hope the reviewer can agree with this position, if not, we can start the training process for more biomarkers.

Comment:
#In Figure 9 it is expected that some information relevant to label classification is lost at the latent dimension is reduced, however, the percentage of instances for which the random inertia is above the label inertia remains relatively stable. From table 3 we can note validation accuracy >0.5, AUROC > 0.5, and F1 score >0.5, meaning that some information is lost but at 2 latent dimensions some relevant information for Ki67 classification remains. A deeper exploration of the information encoded in the latent space that allows for better than random prediction is needed. Examples of supporting data would be dimensionality reduced plots (PCA, UMAP, or tSNE) of the latent space colored by class label side by side with the same scatterplot but with tile images plotted for each point. This should be done for each dimensionality of latent space.

Response:
Indeed, it's interesting to better understand the behavior in the latent space. We added t-sne figures for Ki67, TILS and PR for different dimensions in the supplement.

Comment:
#The data is developed from a multi-centre trial, and interpretation of latent space reduced dimensions could rely on site-specific batch effects (Howard Nature Communications 2021). Please provide baseline model performance for the primary endpoints of interest using relevant meta-data variables as solitary inputs. A model understanding predictive performance of trial site as the only inclusion variable is essential.

Response:
Of course, in general, the sample origin can be a confounding factor, as pointed out by the reviewer. In our cases however, all patients started as Lum B, and the responsive ones become Lum A. The testing for LumA vs LumB is centrally done in IDIBAPS lab in all three treatment related timepoints. We therefore think that this is not an issue in our case.

Comment:
Many figures are of low quality and have text which are either unreadable or very low resolution (example: Figure 5).

Response:
We improved the resolution of the figures.

Comment:
All text should be past tense e.g. [60, 61, 98, 100, 130, 183]

Response:
We reviewed the manuscript and modified the language.

Comment:
Clarification of how tiles are generated, whether they are over the whole tissue section, a pathologist-annotated tumor region, and if white-space is excluded is needed [153-155].

Response:
This is an important point, thank you for the comment. We explained this better and added an explanatory figure Appendix Figure 2.

Comment:
#The trait descriptions can be shorted and would benefit from including the assay used to generate the label.

Response:
We added description on the assay for each trait and shortened the descriptions for some traits.

Please, let us know if there is anything else we should change.

Thank you very much,
Best regards,
Chloé Benmussa
