# Supplementary material for: Latent representation of H&E images retains biological information in a breast cancer cohort
Source: PLoS One. 2025 Sep 25;20(9):e0329221. doi: 10.1371/journal.pone.0329221 (PMC12463230; doi:10.1371/journal.pone.0329221)
Supplement: Attachment — Submitted filename: Response_to_Reviewers_auresp_2.pdf [file pone.0329221.s005.pdf]

Yakhini Research Group
Arazi School of Computer Science
Reichman University

Dear Editors of PLOS ONE,

Thank you for reviewing and handling our manuscript titled "LATENT REPRESENTATION OF H&E IMAGES RETAINS BIOLOGICAL INFORMATION IN A BREAST CANCER COHORT".
Below, please find our response to the comment.

Comment:

"Upon review, we noticed that several of the figures appear blurry and low in resolution, which makes it difficult to interpret the data clearly. Could you please replace all figures with high-quality, high-resolution versions (ideally ≥300 dpi) so that labels, axes, and graphical details are crisp and easily readable?"

Response:

We recreated all the figures to have a resolution of 300 dpi and checked the dimensions of all the images to be in the range of the accepted format.

Since there is no change needed in the manuscript, the files 'Revised Manuscript with Track Changes' and the 'Manuscript' contain the same file, which is the latest version of the Manuscript uploaded to the previous submission.

Please, let us know if there is anything else we should change.

Thank you very much,
Best regards,
Chloé Benmussa
